# A Collusion-Resistant Fingerprinting System for Restricted Distribution of Digital Documents

**DOI:** 10.1371/journal.pone.0081976

**Published:** 2013-12-11

**Authors:** Mario Diego Munoz-Hernandez, Jose Juan Garcia-Hernandez, Miguel Morales-Sandoval

**Affiliations:** Laboratorio de Tecnologias de Informacion, CINVESTAV-IPN, Tamaulipas, Mexico; University of Catania, Italy

## Abstract

Digital fingerprinting is a technique that consists of inserting the ID of an authorized user in the digital content that he requests. This technique has been mainly used to trace back pirate copies of multimedia content such as images, audio, and video. This study proposes the use of state-of-the-art digital fingerprinting techniques in the context of restricted distribution of digital documents. In particular, the system proposed by Kuribayashi for multimedia content is investigated. Extensive simulations show the robustness of the proposed system against average collusion attack. Perceptual transparency of the fingerprinted documents is also studied. Moreover, by using an efficient Fast Fourier Transform core and standard computer machines it is shown that the proposed system is suitable for real-world scenarios.

## Introduction

The adoption of information systems is changing the way organizations work, allowing the automation of their processes and thus making them more efficient. One of the recurring processes of these organizations is the management of the document's lifecycle which is the workflow that defines how users interact with the documents from their creation until their usage in other organization's processes. Currently, many organizations have digitized their physical documents to automate the document's lifecycle which depends on the type of information contained in the digital documents, but has a common flow [1]–[3]:

1. Creation: A user generates a digital document.
2. Review: The digital document is evaluated. Then, it is determined if the digital document is ready to be released.
3. Storage: The digital document is stored in a repository of the organization.
4. Distribution: The digital document can be accessed by authorized users.
5. Usage: Authorized users access and manipulate the digital document.

In this lifecycle, digital documents with sensible information must be available only for authorized users. Cryptography and other security techniques can be used to protect documents during their storage and distribution, but once an authorized user obtains a clear copy document it has no more protection. The authorized user could act dishonestly, by distributing the document to unauthorized entities. The document copy distributed illegally is known as *pirate document* and the user that distributes it is named *traitor*. The problem of pirate documents can continue if it is not possible to identify the traitor [4]–[7]. To solve this problem, invisible watermarking techniques can be used to insert the ID of the user in the document requested. If a pirate copy is detected, it would be possible to identify the traitor by detecting the user's ID, which was inserted when the document was distributed to that user. This way of using the watermarking techniques is known as *digital fingerprinting*.

Digital fingerprinting was originally used to face the illegal distribution of multimedia content such as images [8], audio [9], and video[10], but this technique has been also used to protect digital documents [11], persuading users not to distribute pirate copies and detecting the users who do it. In this study, “fingerprinting” will be used as a synonym for “digital fingerprinting” and “fingerprint” will be used to refer to the unique ID of each user.

For the effectiveness of fingerprinting techniques, it is necessary to satisfy two main properties:

- Perceptual transparency (namely unobtrusiveness, invisibility, or imperceptibility): The original document and its fingerprinted copy must be identical to the user perception. This is measured using the natural language understanding: If the document is entirely legible after the fingerprint insertion, this property is achieved [12], [13].
- Robustness: It is the capacity of user's IDs to survive intentional, and unintentional attacks after being inserted into the document. If the fingerprint is removed or destroyed, the value of the digital document is lost [8].

In an organizational environment, an attack that is prone to happen is a collusion attack. This attack occurs when a set of traitors obtain a copy of a document, each copy having its own fingerprint. Traitors can perform an average of the copies to generate a new pirate document destroying their fingerprints in the process. Traitors who perform a collusion attack are named *colluders*.

### Related Work

Different watermarking insertion techniques have been proposed for digital documents that can be used in fingerprinting. These techniques can be syntactic techniques, semantic techniques, and image-based techniques [12], [14]. In this study, digital documents are represented as images; hence, from these three techniques, image-based techniques are chosen.

The first techniques for watermarking on digital documents represented as images were proposed by Brassil *et al.* [15] and Low *et al.* [16]. They consist of inserting watermarks by shifting the text-lines in a vertical way allowing a bit encode per line. To detect the watermark, the distance between the lines is measured. These techniques only work for formatted documents and the inserted watermark is easy to remove by an averaging collusion attack. In [17], [18], techniques based on horizontally shifting of words in the document were proposed. However, these techniques can only be applied to documents that have variable spacing between adjacent words. The watermark detection is only possible when having the knowledge of the space between words in the original document. Other techniques based on the distance between lines, words, or characters have been proposed in [19]–[25], and most of them have been proved to be robust against indirect attacks such as copying, printing, and scanning. Also, specific schemes have been proposed to face these problems [26], [27]. However, the robustness of these techniques against collusion attacks is not reported.

Spread spectrum techniques for insertion have been widely used for natural images because they are robust for a wide range of attacks, including collusion attacks. Cox *et al.* [8] proposed the first watermarking technique using spread spectrum. In this technique, the user's fingerprint is represented as a spread spectrum sequence that is inserted in the most significant frequency regions of the image, because the less significant regions tend to be discarded when applying filters or other techniques of image processing. When the embedded sequence is extracted, it is necessary to establish a correlation with all the known user sequences to detect the traitor. This strategy increases the detection time linearly, and under a collusion attack it considers that all the users are likely to collude, which is not necessarily true. Despite the spread spectrum being used in [28] and [29] as an insertion technique for digital documents, its resistance to collusion attacks has not been reported.

Wang *et al.* [30] have considered that traitors are more likely to collude with users who share common characteristics such as social circumstances or geographic location. Under this assumption, a hierarchical fingerprinting technique based on spread spectrum has been proposed. Using this technique, users are assigned to groups, and the fingerprint is generated from the user ID and its group ID. At the detection stage, first, the group of colluders is identified and then the users who belong to that group are identified. This reduces the computational cost along with the probability of false-positive detection.

Kuribayashi proposed in [31] a hierarchical fingerprinting scheme based on Code Division Multiple Access (CDMA). In this scheme, users are organized in groups, and the user's fingerprint is represented by a spread spectrum sequence, one for the user ID and another for the group ID. These sequences are orthogonal because they are Discrete Cosine Transform (DCT) basis vectors modulated by a pseudorandom sequence (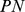) of 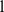 and 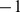 values, allowing retention of orthogonality.

The spread spectrum sequence for a group 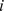 is generated from a vector 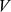 of length 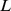 with all entries equal to 0, adding an amount of energy 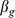 to the entry at position 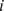. Then, the Inverse DCT (IDCT) is applied to 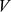 to obtain the 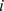-th basis function of the DCT. Finally, 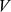 is modulated by a sequence 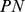 generated from a secret key 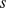, which provides security to the scheme because only the one who knows that key is able to detect groups. The spread spectrum sequence 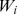 that is generated for the 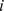-th group is expressed by Equation 1. Each component in the spread spectrum sequence for the group ID can be assigned to a group; therefore, the total amount of groups supported is 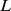. 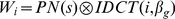
(1)


Generation of the spread spectrum sequence from an user 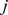 belonging to a group 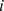 is performed similar to the sequence of the group, with the difference that the 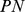 seed is given by the group ID. The spread spectrum sequence assigned to the 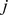-th user is computed according to Equation 2. 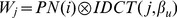
(2)


Using the group ID as the seed of 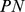, a link is stablished between the group and the user. As the number of groups and users per group is 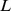, the total amount of users supported is 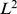. With the spread spectrum sequences of user and group, the fingerprint that represents a user is generated from Equation 3 and the total energy of the fingerprint is obtained from Equation 4. 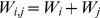
(3)

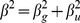
(4)


The generated fingerprint is inserted in the frequency components of the host image. The starting point of insertion denoted as 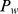 is selected from the low and middle frequencies. When a pirate document is detected, a sequence 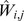 is extracted from its frequency components starting from the point 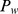, in a nonblinded fashion. From 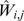, it is possible to obtain a detection sequence 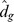 for group IDs, and a detection sequence 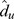 to detect user IDs. Detection is performed using two thresholds, one for the group (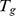) and another for the user (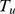). Upon detecting the presence of a group 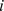, it is possible to detect the users belonging to that group. However, if a user belongs to the group 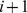, the user will not be detected until detection is performed for that group and the spread spectrum sequences of its users in the image are examined. The system reported in [31] has proved be faster than the previous work as its fingerprint detection strategy is carried out using fast DCT algorithms.

In this study, the performance of the scheme presented in [31] is investigated for digital document distribution applications. Numerous experiments are performed to evaluate the properties of perceptual transparency and robustness. The outline of this paper is as follows: First, experimental results and discussion are presented. In the Methods and Materials section, details of the insertion, detection and determination of thresholds are provided, and the Fast DCT along with the PSNR and SSIM Index metrics used to evaluate perceptual transparency are recalled. Finally, conclusions are provided.

## Results and Discussion

The proposed fingerprinting system implementation must satisfy perceptual transparency and robustness under averaging collusion attacks in digital documents. To achieve that goal, adequate values for the energy of user (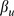), energy of group (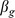), fingerprint length (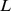), and insertion position (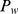) are determined through experimentation. First, values that satisfy perceptual transparency are determined. Then, their best configuration is identified for maximum colluder detection. Simulations of collusion attacks are performed, generating pirate copies of digital documents in TIFF (lossless) and JPEG (lossy) formats. For the experiments, the input documents have been selected from a set of 1000 different digital documents in a JPEG format with average dimensions of 1900×2700 pixels, these documents have been obtained from [32]. Statistical significance is achieved for this sample size as random 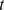-tests results (for significance level equal to 0.05) showed a 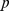-value about 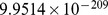 for the biggest.

### Perceptual Transparency

**Figure 1:**
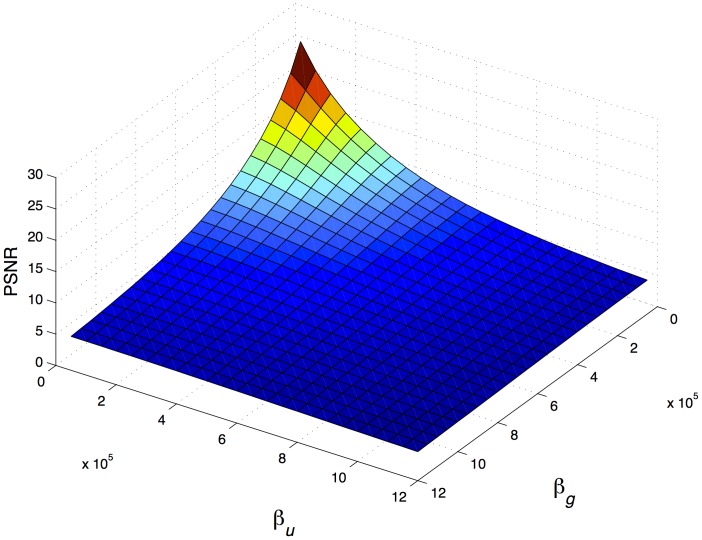
Distortion generated in digital documents due to fingerprint insertion varying the energy assigned to users (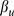) and groups(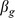), with fixed values of fingerprint length (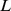) and insertion position (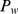). Low PSNR values implies high distortion.

**Figure 2:**
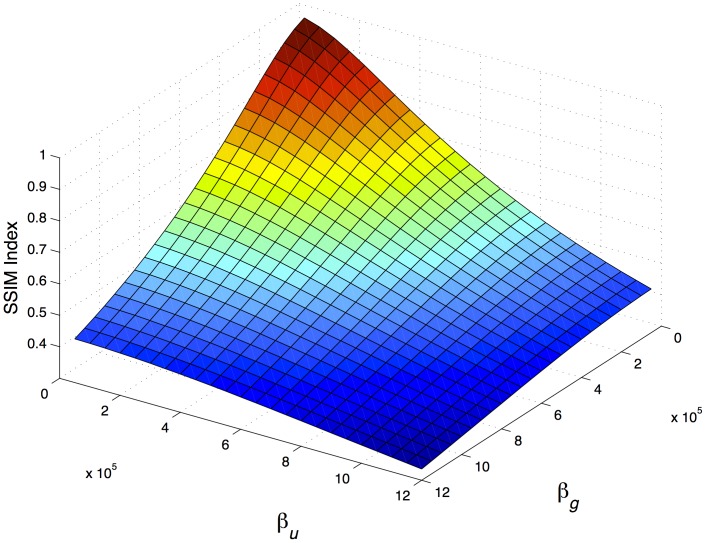
Similarity between an original document and its fingerprinted copy as the energy assigned to users (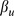) and groups(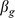) vary, with fixed values of fingerprint length (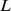) and insertion position (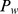). High SSIM Index values implies a high similarity.

To find values for 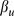, 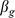, 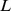, and 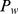 that satisfy perceptual transparency, these values were combined as defined in Table 1 to generate fingerprints, which were inserted in a set of 1000 digital documents. Then, the perceptual transparency was evaluated by using PSNR and SSIM Index as metrics (these metrics are detailed in the Methods and Materials section). It was found that 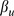 and 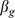 are the factors that affect the perceptual transparency more negatively as their values increase, as shown in Figure 1. Also, it was noticed that lower values of 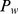 slightly increase the PSNR value, whereas when 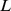 increases, the PSNR value is slightly reduced. By fixing the values of  and 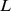 and varying the values of 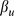 and 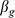, it can be observed in Figures 1 and 2 that the obtained values of PSNR and SSIM Index are correlated. This is consistent with the findings of Hore and Ziou [33].

**Table 1:** Configuration parameters for fingerprint insertion.

Parameter	Initial value	Increment	Maximum value
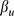	50,000	50,000	1,200,000
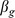	50,000	50,000	1,200,000
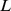	50,000	50,000	1,200 000
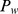	1/6	1/6	5/6

### Inquest Evaluation

As the perceptual transparency for digital documents is achieved by preserving the legibility of the text, an inquest was applied to 100 respondents to determine the lowest PSNR values that satisfied legibility of digital documents. The inquest consisted of the evaluation of a subset having fifteen digital documents with a PSNR value in the range of 4–30 dB. The possible answers available for the respondents were the following:

1. I do not perceive image distortion.
2. I perceive image distortion but the text is easily readable.
3. I perceive image distortion and the text is hardly readable.
4. The text is not readable.

**Figure 3:**
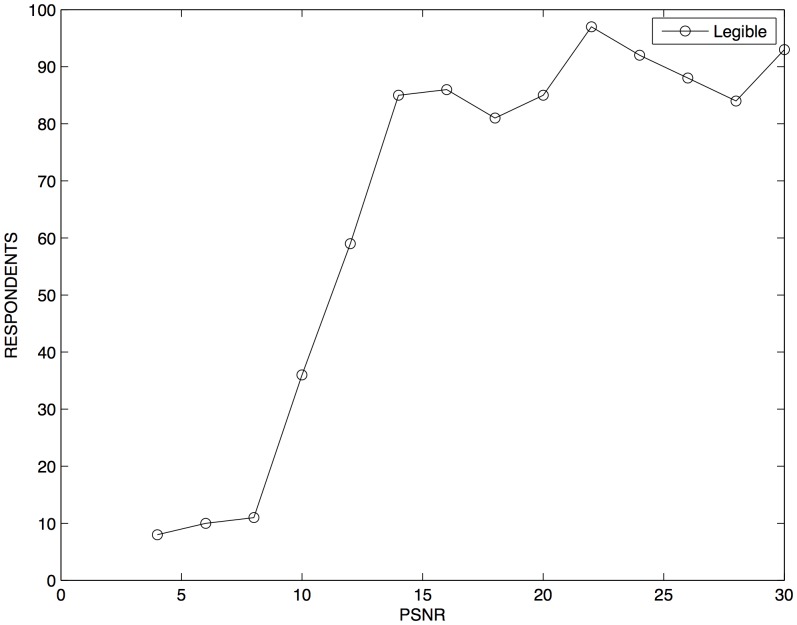
Perception of 100 respondents to fingerprinted digital documents with different PSNR values.

The results of the inquest are shown in Table 2. These results are plotted in Figure 3 that shows the change in the perception of the respondents while the PSNR value in the fingerprinted documents decreases. Most of the respondents considered that digital fingerprinted documents with PSNR values greater than 14 dB and SSIM Index greater than 0.887 are legibles; otherwise, those documents were considered as nonlegibles. Therefore, configurations of 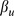, 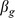, 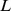, and 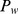 that generate fingerprinted documents with PSNR and SSIM Index equal to or greater than these values satisfy perceptual transparency.

**Table 2:** Perception of respondents to fingerprinted documents with different PSNR and SSIM Index values.

Metrics		Typeofanswer			
PSNR	SSIMIndex	1	2	3	4
30.28dB	0.996	52	41	7	0
28.11dB	0.991	15	69	16	0
26.03dB	0.989	14	74	12	0
24.21dB	0.978	47	45	3	5
22.16dB	0.975	43	55	1	1
20.16dB	0.953	21	64	13	2
18.08dB	0.933	21	58	16	5
16.68dB	0.922	7	79	13	1
14.21dB	0.877	12	73	13	2
12.32dB	0.857	4	55	39	2
10.88dB	0.783	0	36	62	2
8.85dB	0.669	4	7	61	28
6.00dB	0.500	4	6	34	56
4.54dB	0.347	2	6	13	79

### Collusion Attack on Digital Documents in TIFF Format

From the generated set of 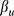, 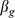, 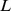, and 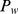, configurations that generate fingerprinted documents were selected with a PSNR value of 16 dB to satisfy perceptual transparency. The selected values of parameters of 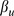, 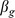, and 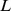 are shown in Table 3. As the best value for 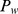 is the lowest one (1/6), this value was selected as the most appropriate and was fixed for the next simulations.

**Table 3:** Configurations of 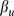, 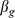 and 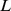 that satisfy perceptual transparency for a fixed 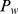.

Configuration	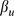	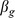	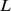
1	200,000	50,000	50,000
2	200,000	100,000	50,000
3	150,000	150,000	50,000
4	100,000	200,000	50,000
5	50,000	200,000	50,000
6	200,000	50,000	100,000
7	200,000	100,000	100,000
8	150,000	150,000	100,000
9	100,000	200,000	100,000
10	50,000	200,000	100,000
11	200,000	50,000	150,000
12	200,000	100,000	150,000
13	150,000	150,000	150,000
14	100,000	200,000	150,000
15	50,000	200,000	150,000
16	200,000	50,000	200,000
17	200,000	100,000	200,000
18	150,000	150,000	200,000
19	100,000	200,000	200,000
20	50,000	200,000	200,000
21	200,000	50,000	250,000
22	200,000	100,000	250,000
23	150,000	150,000	250,000
24	100,000	200,000	250,000
25	50,000	200,000	250,000
26	200,000	50,000	300,000
27	200,000	100,000	300,000
28	150,000	150,000	300,000
29	100,000	200,000	300,000
30	50,000	200,000	300,000
31	200,000	50,000	350,000
32	200,000	100,000	350,000
33	150,000	150,000	350,000
34	100,000	200,000	350,000
35	50,000	200,000	350,000

### Robustness Factors

In Table 3, there are only five configurations of 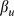 and 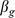 for each value of 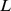 with a PSNR of 16 dB. To determine the values of 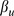 and 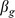 that allow the highest colluder detection probability, the first five configurations of 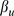 and 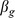 were chosen with 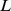 = 50,000. Then, averaging collusion attacks were simulated over 50 digital documents, from 2 colluders to 300 colluders that belong to the same group using the selected values. The fingerprinted documents were generated in the lossless format TIFF with a RGB color scheme.

**Figure 4:**
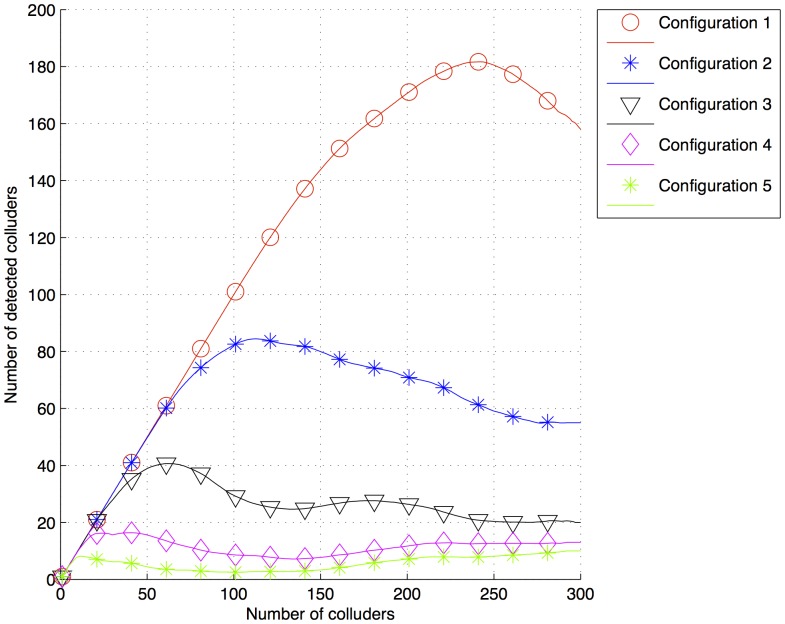
Number of detected colluders after a collusion attack for fingerprints generated with the configurations 1, 2, 3, 4 and 5 in Table 3 and 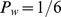
.

**Figure 5:**
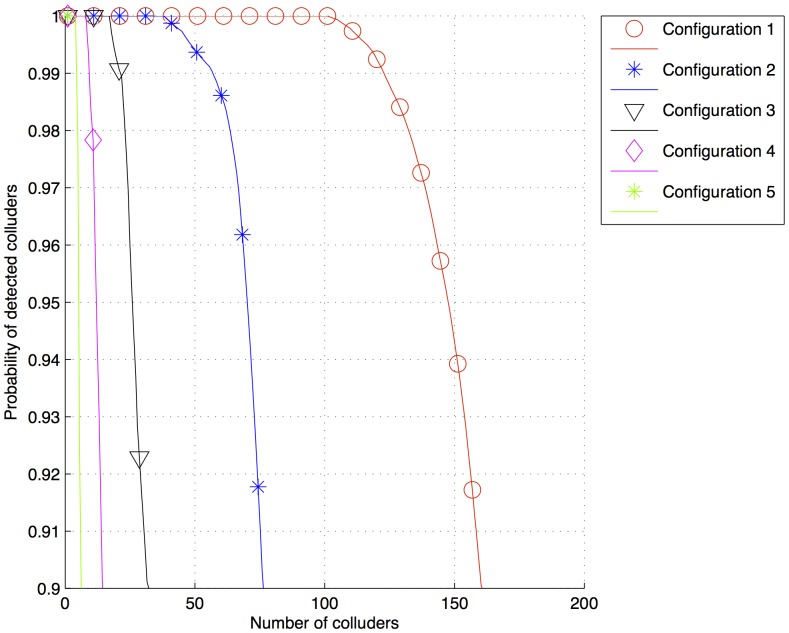
Probability of detected colluders after a collusion attack for fingerprints generated with the configurations 1, 2, 3, 4 and 5 in Table 3 and 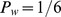
.

**Figure 6:**
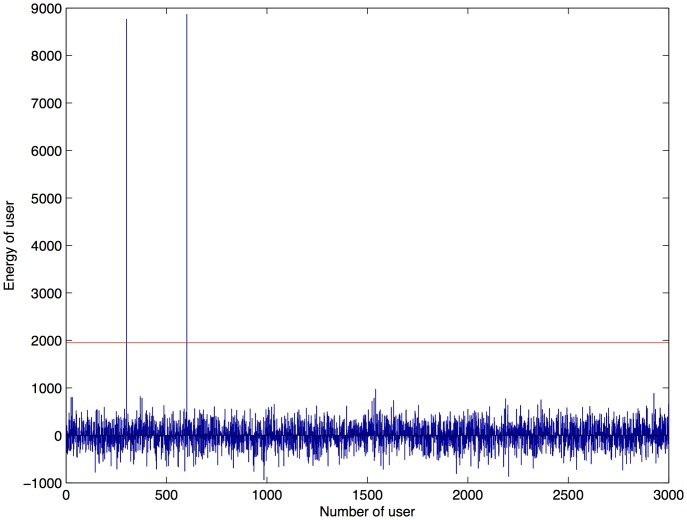
Detection of 2 colluders with IDs 300 and 600, with 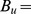
200,000, 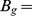
50,000, 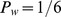 and 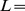
350,000. The red line defines the threshold value.

The results of the simulation are plotted in Figures 4 and 5. Configuration 1 (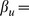
200,000, 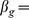
50,000, 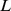 = 50,000) has the highest number of detected colluders, whereas configuration 5 (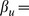
50,000, 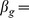
200,000, 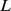 = 50,000) has the lowest one. This is significant because the values of 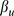 and 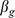 in configurations 1 and 5 are inverted, because in the averaging collusion attack, the value of 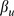 of each colluder is reduced proportionally to the number of colluders. However, as 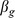 is the same for each user, the energy of 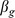 is first accumulated as many times as the number of colluders, and then it is divided by the same value, having no changes. Therefore, the amount of energy assigned to users must be the highest possible to resist a big amount of colluders. Figure 6 shows the detection sequence from a pirate document generated from the collusion of two colluders with ID = 300 and ID = 600. The fingerprints of both the colluders were defined by configuration 1, and despite the reduction in energy, the threshold 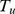 could still detect them. Simulations performed for lossless digital documents presented a considerable amount of noise under 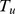. Much of this noise was because of the pixels in the host image after fingerprint insertion, which were not in the range of 0–255. Therefore, negative values were set to 0 and higher values of 255 were set to 255. This rounding off was reflected as noise.

### Fingerprint Length

**Figure 7:**
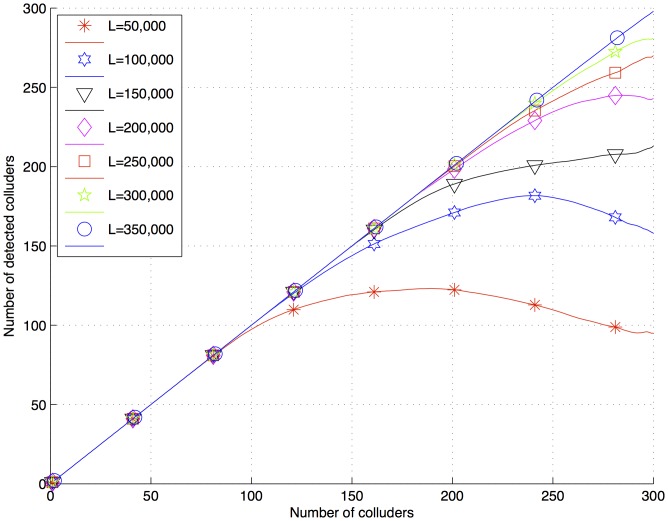
Number of detected colluders after a collusion attack for fingerprints generated with 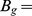
50,000, 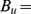
200,000 and 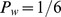.

**Figure 8:**
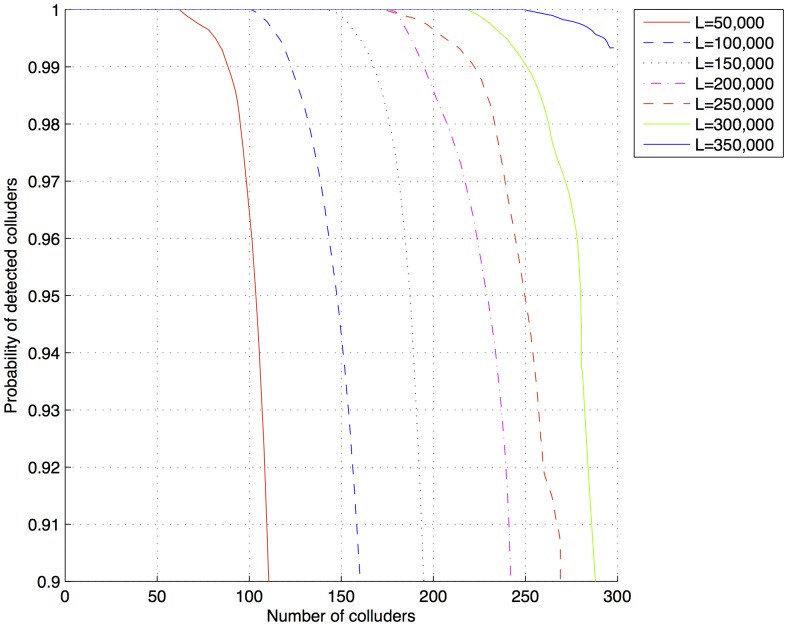
Probability of detected colluders after a collusion attack for fingerprints generated with 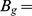
50,000, 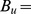
200,000 and 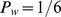.

After defining the values of 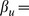
200,000 and 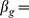
50,000 as the configuration with the highest detection rate, a new simulation of collusion attacks was performed to determine the value of 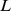 that provides the best detection rate. Configurations with different values of 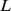, 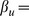
200,000, and 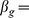
50,000 were selected from Table 3, and these configurations were 1, 6, 11, 16, 21, 26 and 31. The simulation results are plotted in Figures 7 and 8. As the value of 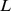 increased, the detection ratio also increased. In [25] Kuribayashi had already mentioned this behavior for images in general, but in that work, it was indicated that the value of 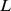 is limited by the image size. It is possible to calculate the highest value of 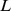 for the document samples, as shown in Equation 5, where 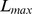 is the maximum value of 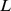, 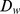 is the document width, 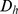 is the document height, 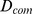 is the number of color components (typically 3), and 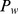 is the insertion point of the fingerprint.
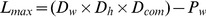
(5)


Using Equation 5, for the sample documents, 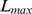 is 12,150,000. Using 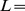
350,000, it was possible to detect 270 colluders in a collusion attack with 300 colluders. Reducing 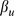 and 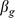 to increase 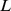 to maintain the perceptual transparency could be a good tradeoff.

### Collusion Attack on Digital Documents in JPEG Format

**Figure 9:**
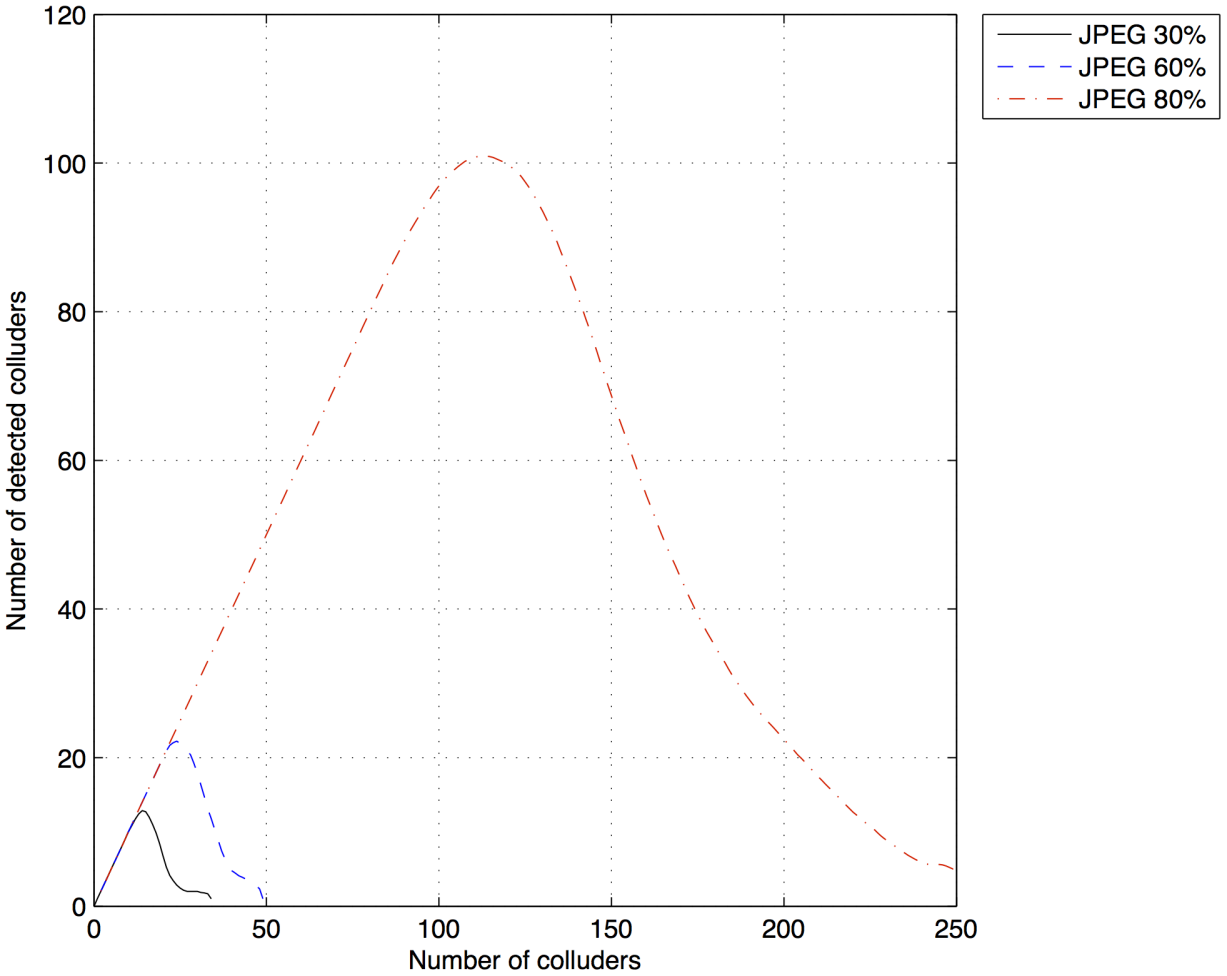
Number of detected colluders after a collusion attack with lossy digital documents for fingerprints generated with 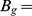
50,000, 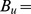
200,000, 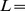
350,000 and 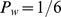.

Once identified that configuration 31 (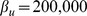, 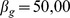 and 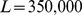) in Table 3 leads to the highest amount of detected colluders, these values were used to generate fingerprints and simulate collusion attacks using the lossy image format JPEG. In these simulations, the fingerprinted digital documents were compressed storing the image in a JPEG format. Then, the collusion attack was simulated and the resulting document was stored again in JPEG format. This implies that the fingerprints in the digital document were affected twofold by the compression process. Figure 9 shows the results of the simulated collusion attacks from 2 to 250 colluders, over 15 digital documents with their quality reduced to 80, 60, and 30%. Comparing the maximum amount of colluders detected using documents in JPEG format (Figure 9) with those using documents in TIFF format (Figure 7), it was observed that the detection rate in documents in JPEG format diminished drastically. However, a considerable amount of colluders were still detected in digital documents in the lossy format JPEG, making this an attractive characteristic for organizations.

### Performance Evaluation

The main functions of the proposed system are the insertion of a fingerprint and its detection. The execution time of these functions are critical when digital documents are fingerprinted in a production environment. To determine if the system holds feasible execution times, performance of insertion and detection of fingerprints were evaluated over 100 digital documents in JPEG and TIFF format (implementation details can be found in the Software Implementation section). The insertion evaluation considers the document read time, the fingerprint insertion time and the fingerprinted document write time. On the other hand, detection evaluation considers the document read time and the fingerprint detection. The results obtained are shown in Table 4. It is noticeable that insertion and detection time does not differ significantly between the formats. The most time-consuming task in the insertion and detection is the execution of the DCT/IDCT, with an execution time higher than 3500 ms. This can be observed in the relation of execution time between the insertion and detection of fingerprints in Table 4; the execution time of the insertion function requires two transformations and is twice the time of detection in which just one transformation is needed. Considerable time is spent in the insertion due to DCT transforms applied over digital documents of large dimensions; however, this time is still feasible for real-life applications. The time for performing the detection of users after the digital document has been read and transformed with the IDCT is very low, around 74 ms, allowing a wide amount of users per group and having an acceptable detection time.

**Table 4:** Performance evaluation of insertion and detection of fingerprints.

Format	Insertion time (read, insert, write)	Detection time (read, detect)	Detection time(detect)
JPEG	7574.14 ms	3764.19 ms	76.53 ms
TIFF	7311.45 ms	3853.06 ms	73.55 ms

### Comparison

In related works (see the Related Work section), techniques for information insertion in digital documents can be divided into line shifting encoding, word shifting encoding, and character space encoding. As mentioned earlier, these techniques have not reported their robustness to collusion attacks. On the other hand, schemes described in [29] and [28] could potentially resist collusion attacks because they are based on spread spectrum techniques. However, these works do not report their robustness to these attacks. It is worth mentioning that for none of these techniques the impact of fingerprint insertion in perceptual transparency has been evaluated. Also, as these insertion techniques have not been conceived originally for fingerprinting, the supported amount of users is not provided. Table 5 shows a comparison of the approach proposed in this work against the approaches reviewed in the Related Work section, where insertion techniques are used on digital documents represented as images. Unlike related works, the selected fingerprinting scheme for the proposed system was validated regarding perceptual transparency and robustness to collusion attacks through experimentation.

**Table 5:** Comparison between the proposed work and other insertion techniques of fingerprinting.

			Robustness			
Work	Domain	Strategy	Collusion-resistant	Lossy compression	Perceptual transparency	Number of users
Low *et al.* [16]	space	line shifting encoding	not reported	yes	not reported	not reported
Low *et al.* [18]	space	line shifting encoding	not reported	yes	not reported	not reported
Alattar [20]	space	line shifting encoding	not reported	yes	not reported	not reported
Kim *et al.* [21]	space	word shifting encoding	not reported	yes	not reported	not reported
Yawai *et al.* [25]	space	word shifting encoding	not reported	yes	not reported	not reported
Huang *et al.* [22]	space	character space encoding	not reported	yes	not reported	not reported
Chotikakamthorn [19]	space	character space encoding	not reported	yes	not reported	not reported
Qadir *et al.* [28]	frequency	spread spectrum	not reported	not reported	not reported	not reported
He *et al.* [29]	frequency	spread spectrum	not reported	yes	not reported	not reported
Proposed	frequency	spread spectrum	yes	yes	guarantee	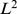

## Materials and Methods

This section describes the Fast DCT and Inverse DCT (IDCT) Algorithms as well as the fingerprint insertion and detection methods. Also, the PSNR and SSIM Index metrics used for evaluation of the perceptual transparency in the proposed system are described.

### Fast DCT and Inverse DCT Algorithms

It is known that the Fourier transform of a real-even function 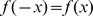 is real-even, and 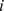 times the Fourier transform of a real-odd function 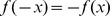 is real-odd; thus, for these symmetry conditions, it is not necessary to use complex inputs/output. Therefore, it is possible to compute the DCT or the Discrete Sine Transform (DST) by utilizing an FFT algorithm.

Let the input vector 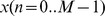 be even around 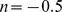 and even around 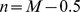; it is possible to show that DFT(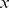) is the nonnormalized DCT of 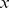, 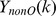 described as follows:
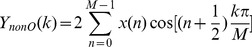
(6)with basis:
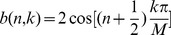
(7)


The basis set described in Equation (7) is nonorthogonal; therefore, it is necessary to normalize Equation (6) to get the orthogonal transform as follows:
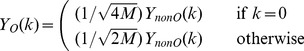
(8)


On the other hand, let the input vector 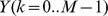 be even around 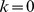 and odd around 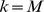; it is possible to show that DFT(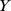) is the nonnormalized Inverse DCT of 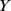, 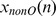 described as follows:
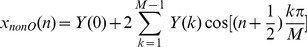
(9)


As shown in Equation (6), a normalization procedure is necessary to obtain the orthogonal transform. The normalization is carried out as follows: 
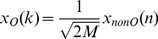
(10)


In the literature, fast algorithms for the DFT have been extensively reported and very efficient software libraries exist [34]. In this work, these libraries are utilized as a module of DCT computing, reducing the effort required for efficient implementation to a normalization stage implementation for DCT.

### Fingerprinting Method

The fingerprinting method for digital images consists in two procedures (the fingerprint insertion and detection) as follows:

#### Fingerprint Insertion Method

**Figure 10:**
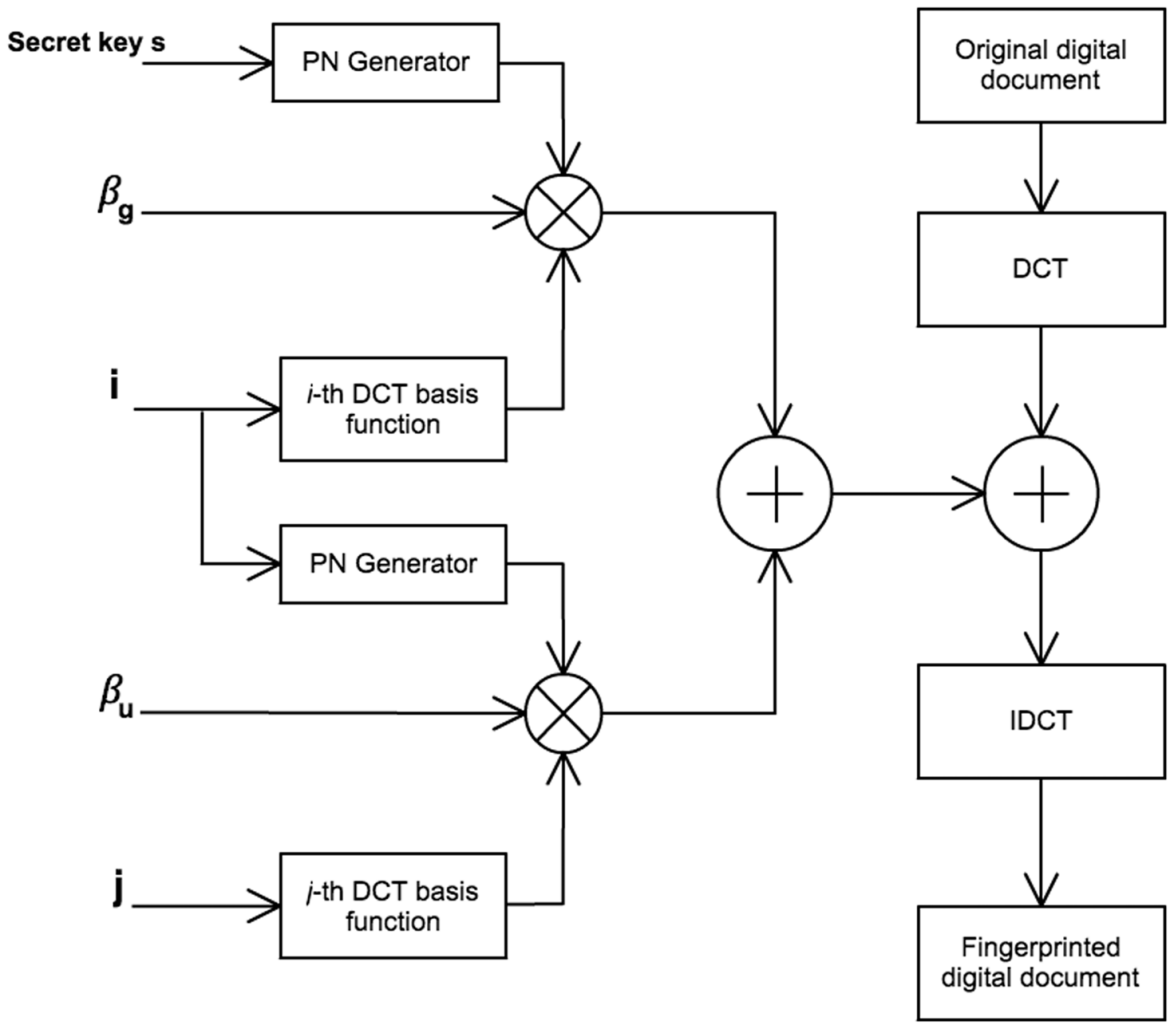
Diagram of fingerprint insertion method.

The following steps describe the insertion method of the fingerprint 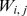 described in Equation 1. This method is graphically shown in Figure 10:

1. Transform the image to the frequency domain using the DCT function.
2. Select 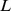 coefficients of the low and middle frequencies from a position 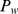. The selected coefficients are denoted as: 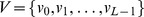
(11)
3. Insert the fingerprint additively in the extracted coefficients: 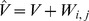
(12)
4. Transform the image to the spacial domain using the Inverse DCT function to get the fingerprinted image.

#### Fingerprint Detection Method

The following steps describe the detection of the user's fingerprint in an illicit copy of an image representing a digital document:

1. Transform the illicit copy to the frequency domain using the DCT function.
2. Select 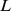 coefficients of the low and middle frequencies from the position 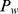. The selected coefficients are denoted as: 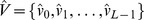
(13)
3. Detect the group ID:
  - Generate 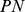 using the secret key 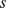.
  - Use the DCT function to extract the detection sequence 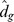: 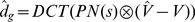
(14)
  - Calculate the variance 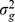 of 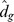 considering the probability distribution and determine the threshold 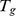 from a given false-positive denoted as 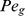: 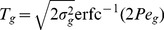
(15)where 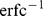 stands for the inverse complementary error function defined in Equation 16 and 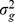 is computed as shown in Equation 17.
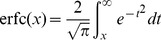
(16)

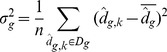
(17)
  - If 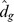 in the input 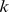 exceeds the threshold 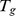, it is determined whether 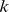 is the group ID.
4. Detect the user ID:
  - Generate 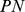 using the ID 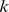 of the detected group.
  - Use the DCT function to extract the detection sequence 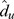: 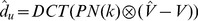
(18)
  - Calculate the variance 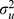 of 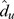 in a similar way as in Equation 17, considering the probability distribution and determine the threshold 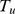 from a given false-positive denoted as 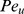:
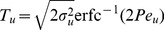
(19)
  - If 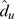 in the input 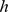 exceeds the threshold 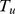, it is determined that 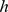 is the user ID.

**Figure 11:**
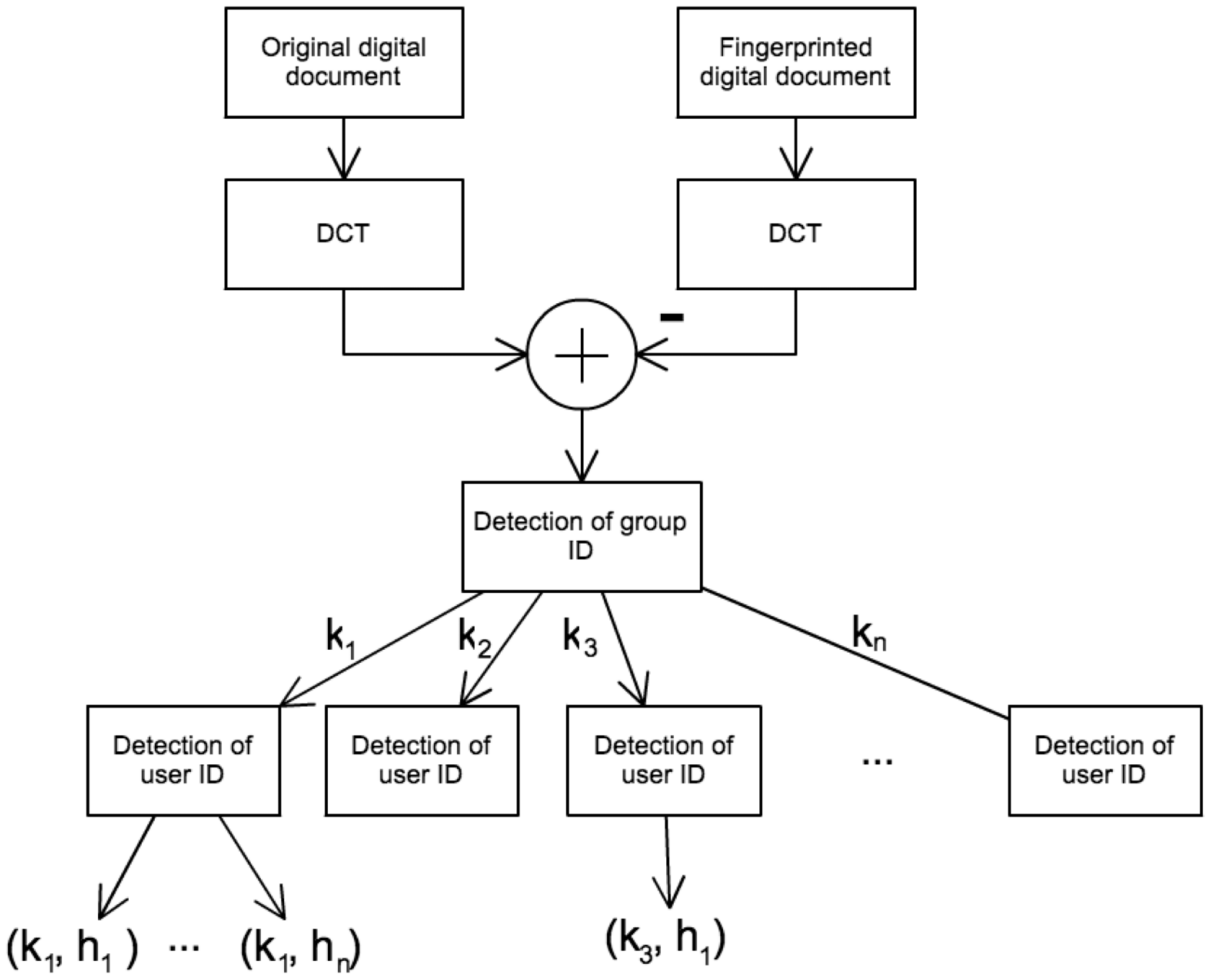
Diagram of fingerprint detection method.

This hierarchical detection of fingerprints is illustrated in Figure 11.

### Evaluation of Perceptual Transparency

In this study, two methods for evaluation of the perceptual transparency of fingerprinted images were utilized. Those methods are described as follows:

#### Peak Signal-to-Noise Ratio

Peak Signal-to-Noise Ratio (PSNR) measures the similarity of two images. It defines the relation between the maximum energy of a signal and the noise that affects expressing this difference in decibels [33], [35]. Given an 8-bit grayscale image 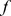 and a copy of the altered image 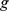, both of size 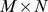, the PSNR between 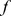 and 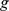 is defined by:
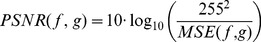
(20)

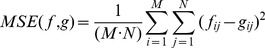
(21)


For Mean Square Error (MSE), the difference between pixels 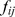 and 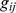 is considered as an error that generates image quality loss. As MSE tends to zero, the value of PSNR approaches infinity. The higher the PSNR (f, g) values, the higher the image quality.

#### Structural Similarity Index

Structural Similarity Index (SSIM) is a particular implementation of the structural similarity philosophy [33], and it is considered correlated with the human visual system [36]. For two image signals 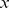 and 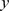, comparison of three components: luminance, contrast, and structure, is necessary. These components are relatively independent because object structures in images neither depend on illumination nor contrast. The luminance is defined by the function in Equation 22, where 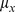 is the standard deviation of 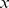. 
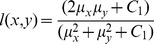
(22)


Then, the closeness of the contrast of the images is measured as shown in Equation 23, where 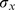 is the variance of 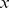. 
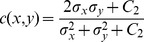
(23)


The structure comparison is defined by Equation 24, where 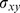 is the covariance between 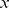 and 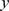. 
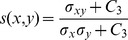
(24)


Finally, the three components are combined to get the overall similarity measure expressed in Equation 25, where the exponents 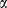, 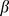, and 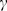 are positive integers that define the importance of each component. 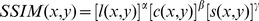
(25)


### Software Implementation

The fingerprinting system was implemented in C++ language using the GCC compiler version 4.2.1 with Ubuntu 12.10 as operative system, an Intel Core i5 processor at 2.7 GHz and 4 GB RAM. To perform the DCT and IDCT Transform, the FFTW library version 3.3.3 was used [34]. To read and write TIFF images, the libtiff version 3.6.1 was used [37], and for the JPEG image, the jpeglib version 8.0 was used [38]. For computing the PSNR and SSIM Index value the IQA library version 1.1.2 was used[39]. Finally, the source code is available upon request.

## Conclusions

In this study, the collusion-resistant fingerprinting method proposed by Kuribayashi has been implemented in the context of restricted distribution of digital documents. The performance of the system was evaluated by defining permissible levels of distortion in terms of legibility of fingerprinted documents, and appropriated values of parameters have been determined to achieve higher colluder detection. Estimation of the required computing time for insertion and detection of fingerprints in digital documents was also carried out. For the fingerprinting scheme, it was shown that the energy assigned to users must be higher than that assigned to groups to have a higher colluder detection probability. Furthermore, an equation to determine the maximum fingerprint's length was proposed. The system could fully detect up to 270 of 300 colluders for lossless compressed digital documents. The detector performance after lossy compression remains competitive for real-work environments. The number of users available and the high-quality lossy compression robustness make the proposed system suitable for implementation in a production environment.

## References

[1] Adobe (2010) Making the case PDF/A and Adobe Acrobat. White paper, Adobe Systems Incorporated., Available: http://goo.gl/F4ZkL - Accessed 2013 Jun 22.

[2] Gupta R, Karayil A, Rajendran R (2008) Contract lifecycle management. White paper, Infosys, Available: http://goo.gl/aEQj9 - Accessed 2013 Jun 22.

[3] Villars SFRL (2006) The information lifecycle management imperative. White paper, IDC Information and data, Available: http://goo.gl/4aOqo - Accessed 2013 Jun 22.

[4] Berghel H (2012) Wikileaks and the matter of private manning. Computer 45: : 70–73.

[5] Coles-Kemp L, Theoharidou M (2010) Insider threat and information security management. In:Probst CW, Hunker J, Gollmann D, Bishop M, editors, Insider Threats in Cyber Security, Springer US, volume 49 of *Advances in Information Security*. pp.45–71.

[6] Kessler G (2012) Information security: New threats or familiar problems? Computer 45: : 59–65.

[7] Gaff BM, Loren RA, Spinney EA (2012) Intellectual Property, Part II. Computer 45: : 9–11.

[8] Cox I (1997) Secure spread spectrum watermarking for multimedia. IEEE Transactions on Image Processing 6: 1673–1687. 18285237 10.1109/83.650120

[9] Garcia-Hernandez JJ, Feregrino-Uribe C, Cumplido R (2013) Collusion-resistant audio fingerprinting system in the modulated complex lapped transform domain. PLoS ONE 8: e65985. 23762455 10.1371/journal.pone.0065985 PMC3675215

[10] Cox IJ, Kilian J, Leighton T, Shamoon T (1996) Secure spread spectrum watermarking for images, audio and video. In: International Conference on Image Processing, 1996. Proceedings. volume 3 , pp. 243–246.

[11] Schick R, Ruland C (2011) Document tracking - on the way to a new security service. In: 2011 Conference on Network and Information Systems Security (SAR-SSI). pp. 1–5.

[12] Jalil Z, Mirza A (2009) A review of digital watermarking techniques for text documents. In: International Conference on Information and Multimedia Technology. ICIMT '09. pp. 230–234.

[13] Zhou X, Wang Z, Zhao W, Wang S, Yu J (2009) Performance analysis and evaluation of text watermarking. In: International Symposium on Computer Network and Multimedia Technology. pp. 1–4.

[14] Zhou X, Zhao W,Wang Z, Pan L (2009) Security theory and attack analysis for text watermarking. In: International Conference on E-Business and Information System Security. EBISS '09. pp. 1–6.

[15] Brassil JT, Member S, Low S, O'Gorman L, Maxemchuk NF (1995) Electronic marking and identification techniques to discourage document copying. In: IEEE Journal on Selected Areas in Communications. pp. 1278–1287.

[16] Low S, Maxemchuk N, Lapone A (1998) Document identification for copyright protection using centroid detection. IEEE Transactions on Communications 46: : 372–383.

[17] Brassil J, Low S, Maxemchuk NF, O'Gorman L (1994) Hiding information in document images. In: Conference on Information Sciences and Systems (CISS-95). pp. 482–489.

[18] Low S, Maxemchuk N, Brassil J, O'Gorman L (1995) Document marking and identification using both line and word shifting. In: INFOCOM '95, Proceedings of the Fourteenth Annual Joint Conference of the IEEE Computer and Communication Societies. pp. 853–860 vol.2.

[19] Chotikakamthorn N (1998) Electronic document data hiding technique using inter-character space. In: The 1998 IEEE Asia-Pacific Conference on Circuits and Systems. IEEE APCCAS 1998. pp. 419–422.

[20] Alattar AM, Alattar OM (2004) Watermarking electronic text documents containing justified paragraphs and irregular line spacing. In: Delp EJ, Wong PW, editors, Security, Steganography, and Watermarking of Multimedia Contents. SPIE, volume 5306 of Proceedings of SPIE, pp. 685–695.

[21] Kim YW, Moon KA, Oh IS (2003) A text watermarking algorithm based on word classification and inter-word space statistics. In: Proceedings Seventh International Conference on Document Analysis and Recognition 2003. pp. 775–779.

[22] Huang D, Yan H (2001) Interword distance changes represented by sine waves for watermarking text images. IEEE Transactions on Circuits and Systems for Video Technology 11: 1237–1245.

[23] Low SH, Maxemchuk NF (2000) Capacity of text marking channel. IEEE Signal Processing Letters 7: 345–347.

[24] Choo HG, Kim WY (2004) Data-hiding capacity improvement for text watermarking using space coding method. In: Kalker T, Cox I, Ro Y, editors, Digital Watermarking, Springer Berlin Heidelberg, volume 2939 of Lecture Notes in Computer Science. pp. 593–599.

[25] Yawai W, Hiransakolwong N (2012) Increase the hiding-bit capacity and strength for text watermarking with the line intersection on text image. In: 8th International Conference on Computing Technology and Information Management (ICCM). volume 1, pp. 427–433.

[26] Tang YL, Huang YT (2010) Print-and-scan resilient watermarking for authenticating paper-based certificates. In: 2010 First International Conference on Pervasive Computing Signal Processing and Applications (PCSPA). pp. 357–361.

[27] Lefebvre F, Gueluy A, Delannay D, Macq B (2001) A print and scan optimized watermarking scheme. In: 2001 IEEE Fourth Workshop on Multimedia Signal Processing. pp. 511–516.

[28] Qadir M, Ahmad I (2005) Digital text watermarking: secure content delivery and data hiding in digital documents. In: 39th Annual 2005 International Carnahan Conference on Security Technology, 2005. CCST '05. pp. 101–104.

[29] He B, Wu Y, Kang K, Guo W (2009) A robust binary text digital watermarking algorithm for print-scan process. In: 2009 WRI World Congress on Computer Science and Information Engineering. volume 7, pp. 290–294.

[30] Wang ZJ, Wu M, Trappe W, Liu KJR (2004) Group-oriented fingerprinting for multimedia forensics. EURASIP Journal on Applied Signal Processing 2004: 2153–2173.

[31] Kuribayashi M (2011) Hierarchical spread spectrum fingerprinting scheme based on CDMA technique. EURASIP Journal on Information Security 2011: 16.

[32] de Madrid A (2013). Biblioteca Digital del Ateneo de Madrid. Available: http://goo.gl/eioo6. - Accessed 2013 Jun 22.

[33] Hore A, Ziou D (2010) Image quality metrics: PSNR vs. SSIM. In: 20th International Conference on Pattern Recognition (ICPR). Washington, DC, USA, pp. 2366–2369.

[34] Frigo M, Johnson S (2005) The design and implementation of fftw3. Proceedings of the IEEE 93: 216–231.

[35] Ismail Avcbas BS (1999) Statistical analysis of image quality measures. Technical report, Department of Electrical and Electronic Engineering, Bogaziçi University, İstanbul, Turkey.

[36] Wang Z, Bovik A, Sheikh H, Simoncelli E (2004) Image quality assessment: from error visibility to structural similarity. IEEE Transactions on Image Processing 13: 600–612. 15376593 10.1109/tip.2003.819861

[37] Frank Warmerdam MW Andrey Kiselev, Kelly D (2013). Libtiff. Available: http://www.libtiff.org/. - Accessed 2013 Jun 22.

[38] IJG (2013). Libjpeg. Available: http://www.ijg.org/. - Accessed 2013 Jun 22.

[39] Distler T (2013). IQA. Available: http://tdistler.com/iqa/. - Accessed 2013 Jun 22.

